# Diagnostic performance of serum blood urea nitrogen to creatinine ratio for distinguishing prerenal from intrinsic acute kidney injury in the emergency department

**DOI:** 10.1186/s12882-017-0591-9

**Published:** 2017-05-25

**Authors:** Guillaume Manoeuvrier, Kalyane Bach-Ngohou, Eric Batard, Damien Masson, David Trewick

**Affiliations:** 1 Department of Medicine, Clinique Jules Verne, Nantes, France; 2 Department of Biology, Laboratory of Clinical Biochemistry, CHU Nantes, Nantes, France; 3 Department of Emergency Medicine, CHU Nantes, Nantes, France; 4 Service des Urgences, CHU Hotel Dieu, 44000 Nantes, France

**Keywords:** Acute kidney injury (AKI), Blood urea creatinine ratio (BCR), Diagnostic performance, Emergency department, Prerenal acute kidney injury

## Abstract

**Background:**

The blood urea nitrogen to creatinine ratio (BCR) has been used since the early 1940s to help clinicians differentiate between prerenal acute kidney injury (PR AKI) and intrinsic AKI (I AKI). This ratio is simple to use and often put forward as a reliable diagnostic tool even though little scientific evidence supports this. The aim of this study was to determine whether BCR is a reliable tool for distinguishing PR AKI from I AKI.

**Methods:**

We conducted a retrospective observational study over a 13 months period, in the Emergency Department (ED) of Nantes University Hospital. Eligible for inclusion were all adult patients consecutively admitted to the ED with a creatinine >133 μmol/L (1.5 mg/dL).

**Results:**

Sixty thousand one hundred sixty patients were consecutively admitted to the ED. 2756 patients had plasma creatinine levels in excess of 133 μmol/L, 1653 were excluded, leaving 1103 patients for definitive inclusion.

Mean age was 75.7 ± 14.8 years old, 498 (45%) patients had PR AKI and 605 (55%) I AKI. BCR was 90.55 ± 39.32 and 91.29 ± 39.79 in PR AKI and I AKI groups respectively. There was no statistical difference between mean BCR of the PR AKI and I AKI groups, *p* = 0.758. The area under the ROC curve was 0.5 indicating that BCR had no capacity to discriminate between PR AKI and I AKI.

**Conclusions:**

Our study is the largest to investigate the diagnostic performance of BCR. BCR is not a reliable parameter for distinguishing prerenal AKI from intrinsic AKI.

## Background

The blood urea nitrogen to creatinine ratio (BCR) has been used since the early 1940s to help clinicians differentiate between prerenal acute kidney injury (PR AKI) and intrinsic AKI (I AKI) [1]. This ratio is simple to use and is often put forward as a reliable diagnostic tool. Indeed many textbooks of internal medicine, nephrology and critical care continue to advocate the use of BCR even though its usefulness in the diagnosis and clinical management of AKI remains unclear [2–4].

Under normal conditions, BCR is less than 100 (with urea and creatinine concentrations expressed in mmol/L) [2–5]. In states of renal hypoperfusion with intact tubular function, blood urea nitrogen (BUN) is considered to rise out of proportion to plasma creatinine concentration, due to avid urea reabsorption by the proximal tubule, the BCR typically becoming >100 [6].

One of the first to put forward this tool was Fishberg in 1939 when he observed that “*an increase in urea content of the blood may be considerable before the creatinine value rises in prerenal azotemia”* [1]. However, as soon as 1947, other investigators found no such relationship [7]. Since then, very few studies (human or animal) have addressed the question and their results are conflicting [8–11]. These studies consisted of small series of patients, essentially from intensive care units, the largest of which included only 103 patients and this study was not specifically aimed at investigating BCR [11].

AKI is common in Emergency Departments (ED) [12]. It can be challenging to differentiate prerenal from intrinsic acute kidney injury in this setting. Indeed, the ED physician has a short time frame to make decisions, he may not have access to current medication or baseline creatinine, have incomplete medical history and doesn’t have by definition the responsiveness to a fluid challenge.

The BCR is one of the diagnostic tools recommended to ED physicians for differentiating between PR and I AKI, yet it has never been specifically studied in this setting [3].

The aim of this study was to determine whether BCR is a reliable parameter for distinguishing prerenal from intrinsic AKI in a population of patients admitted to hospital via the ED.

## Methods

### Study design and patient selection

We conducted a retrospective observational study over a 13 month period, from 1st of November 2013 to the 30th of November 2014, in the Medical Emergency Department of Nantes University Hospital. Trauma patients were not admitted to this unit. The need for informed consent was waived by the institutional review board of Nantes University Hospital because of the anonymous and purely observational nature of the study.

Eligible for inclusion were all adult patients (≥ 16 years old) consecutively admitted to the ED with a creatinine >133 μmol/L (1.5 mg/dL). Patients were excluded if on chronic dialysis or had a prior kidney transplant, stable chronic kidney disease, obstructive AKI, length of hospital stay less than 48 h, no follow up creatinine during the following 7 days of hospitalisation. If a patient was admitted to the ED on several occasions during a 7 day period, only the first creatinine >133 μmol/L was taken into account, the other admissions were excluded. Patients with no baseline creatinine (lowest creatinine measured 12 months before or after hospital admission) were also excluded.

### Laboratory techniques

Blood samples were collected and centrifuged at 2000 g for 10 min at 4 °C within 1 h after venipuncture. All biochemical measurements of urea and creatinine were performed in the same laboratory (Laboratory of Clinical Biochemistry, University Hospital of Nantes) respectively with an enzymatic kinetic UV assay and a kinetic colorimetric assay based on the Jaffé method on Cobas c701 (Roche Diagnostics, Mannheim) according to the manufacturer’s instructions.

### Study definitions

We defined AKI according to “Kidney Disease: Improving Global Outcomes” (KDIGO) classification scheme based on changes in plasma creatinine during the 7 days after admission (Table 1) [13]. Prerenal AKI (PR AKI) was defined as non-obstructive AKI with a return of plasma creatinine to 110% (or less) of baseline value during the 7 days following admission. The baseline value of plasma creatinine was the lowest creatinine measured 12 months before or after hospital admission. Intrinsic AKI (I AKI) was defined as non-obstructive AKI that didn’t meet the criteria of pre-renal AKI.

**Table 1:** Staging of Acute Kidney Injury (AKI) according to KDIGO criteria

Stage	Criteria
Stage 1	One of the following • Serum creatinine increased 1.5–1.9 times baseline
	• Serum creatinine increase >26.5 μmol/L (0.3 mg/dL)
	• Urinary output <0.5 mL/kg/h for 6–12 h
Stage 2	• Serum creatinine increase 2.0–2.9 times baseline
	• Urinary output <0.5 mL/kg/h for more than 12 h
Stage 3	• Serum creatinine increase >3 times baseline
	• Serum creatinine increases to >353.6 μmol/L (4.0 mg/dL)
	• Initiation of renal replacement therapy
	• Urinary output <0.3 mL/kg/h during more than 24 h
	• Anuria for more than 12 h

### Chart review

Patient’s records were reviewed using the hospital admissions and discharges database. Relevant data was entered in a format that could be converted to an Excel spreadsheet for analysis. The following information was obtained for each admission: basic demographics, dates of hospital admission and discharge, admission plasma creatinine, blood urea nitrogen (BUN) levels and BCR, information concerning urinary obstruction, lowest creatinine measured 12 months before/after admission, lowest creatinine measured during the 7 days following admission and presence or absence of AKI using KDIGO classification.

### Statistical analysis

All statistical analysis was performed using GraphPad Prism Software (La Jolla, CA, USA). Quantitative data were expressed as mean ± standard deviation (SD). Comparison of biochemical and clinical data between prerenal AKI (PR AKI) and intrinsic AKI (I AKI) patients was done using unpaired t-tests. Predictive performance of BCR in terms of specificity and sensitivity was performed using Receiver Operating Characteristic (ROC) analysis. For all analyses, a *P* value <0.05 was considered to be statistically significant.

## Results

**Fig. 1:**
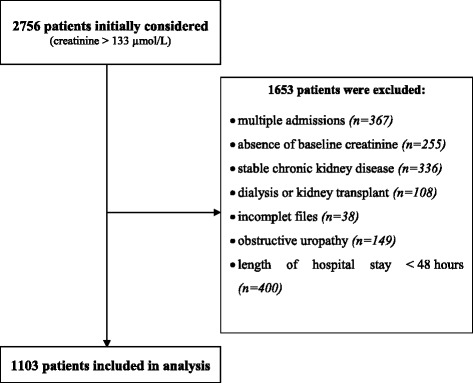
Study Flow-chart – Patients admitted with plasma creatinine >133 μmol/L to the Medical Emergency Department of Nantes University Hospital from 1st of November 2013 to the 30th of November 2014

During the study period, 60,160 patients ≥16 years old were consecutively admitted to the Emergency Department of Nantes University Hospital. Creatinine levels were measured 28,149 times for 26,299 patients. Two thousand seven hundred and fifty six had plasma creatinine levels in excess of 133 μmol/L but 1653 were excluded because they met one or more exclusion criteria, leaving 1103 patients for definitive inclusion (Fig. 1).

The demographic characteristics of the 1103 included patients are presented in Table 2. Mean age was 75.7 ± 14.8 years old (range [16–103]; median 80), 693 (62.8%) were male. According to our definitions, 498 (45%) patients had prerenal AKI (PR AKI) and 605 (55%) intrinsic AKI (I AKI). The PR AKI and I AKI groups do not differ in terms of age (*p* = 0.518) (Table 2). Patients are classified according to their stage of AKI in Table 3.

**Table 2:** Demographic and biological characteristics of patients with intrinsic AKI (I-AKI) and prerenal AKI (PR-AKI). Results are presented as number of patients and proportions when appropriate or means (± standard deviation)

	I-AKI (*N* = 605)	PR-AKI (*N* = 498)	P
Age (years)	75.7 (14.3)	75.7 (15.3)	0.518
Gender, n male (%)	370 (61.2)	323 (64.9)	
Plasma creatinine at admission (μmol/L)	232.1 (124.8)	209.6 (118.3)	0.002
Lowest plasma creatinine during the 7 days following admission (μmol/L)	162.4 (96.6)	118.0 (65.2)	<0.001
Blood urea nitrogen at admission (mmol/L)	19.8 (9.6)	18.1 (9.5)	0.003
Blood urea nitrogen to creatinine ratio at admission (mmol/L/mmol/L)	91.3 (39.8)	90.6 (39.3)	0.758

**Table 3:** Staging of AKI patients according to KDIGO criteria

	Stage 1	Stage 2	Stage 3
Number of AKI patients (%)	334 (30.3)	576 (52.2)	193 (17.5)
PR AKI (%)	58.7	42.5	29.5
I AKI (%)	41.3	57.5	70.5

**Fig. 2:**
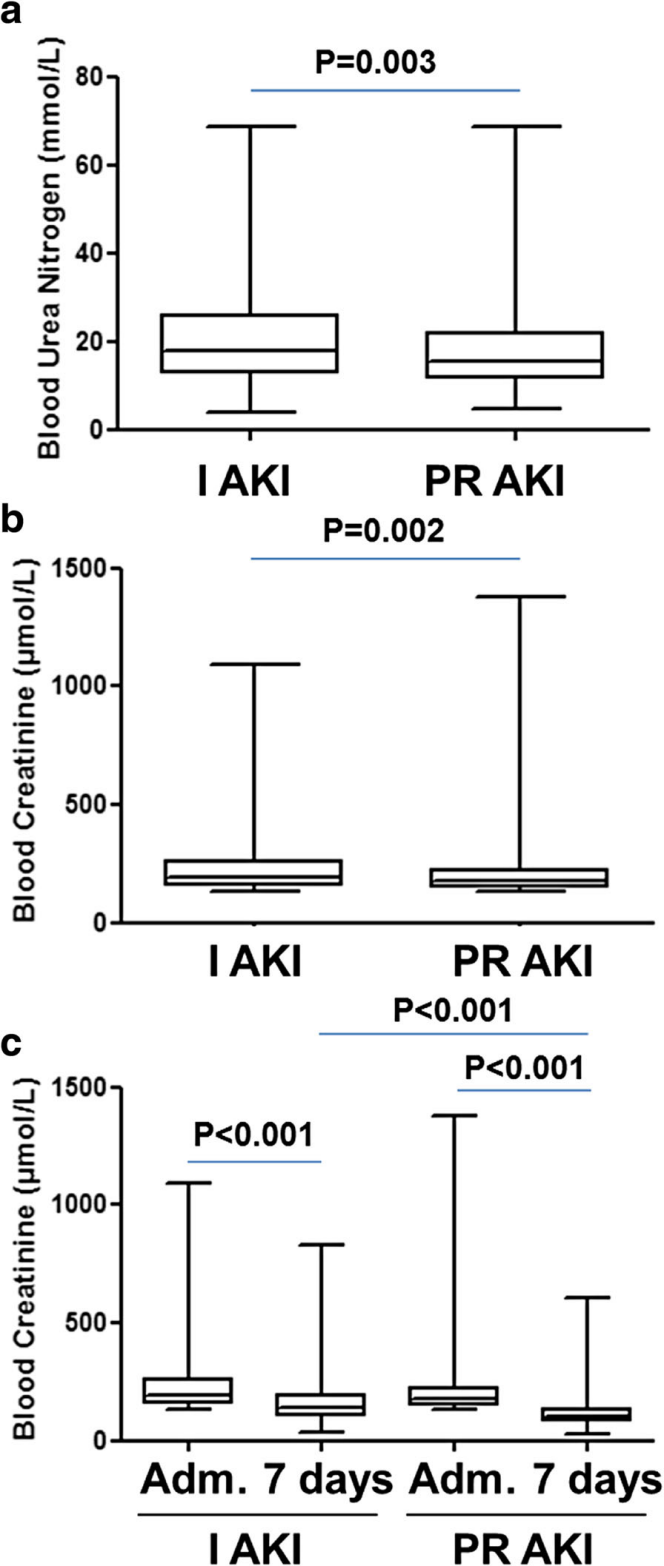
Distribution of blood urea nitrogen (BUN), plasma creatinine values according to AKI groups. The box extends from the 25th to 75th percentiles. The line in the middle of the box is plotted at the median and whiskers delimit min to max values. **a** - BUN values at admission of patients with prerenal (PR AKI) and intrinsic AKI (I AKI); (**b)** - Plasma creatinine values at admission of patients with PR AKI and I AKI; (**c**) - Lowest plasma creatinine during the 7 days following admission (Adm.) of patients with PR AKI and I AKI

At admission, mean blood urea nitrogen (BUN) concentration was 18.1 ± 9.5 mmol/L (range [4.8–68.6]; median 15.6) and 19.8 ± 9.6 mmol/L (range [4.0–68.7]; median 15.9) in PR AKI and I AKI groups respectively (*p* = 0.003) (Fig. 2a and Table 2). At admission, mean plasma creatinine concentration was 209.6 ± 118.3 μmol/L (range [134–1376]; median 177) and 232.1 ± 124.8 μmol/L (range [134–1089]; median 190) in the PR AKI and I AKI groups respectively (*p* = 0.002) (Fig. 2b and Table 2).

During the 7 days following admission, the patient’s lowest plasma creatinine concentrations were selected and compared with their baseline creatinines (Fig. 2c and Table 2). In the PR AKI group, the mean lowest creatinine concentrations during the 7 days follow-up was 118.0 ± 65.2 μmol/L (range [30–606]; median 105). The mean difference with plasma creatinine concentrations at admission was 91.6 μmol/L. In the intrinsic AKI group, the mean lowest creatinine concentrations during the 7 days follow-up was 162.4 ± 96.6 μmol/L (range [32–832]; median 138). The mean difference with plasma creatinine concentrations at admission was 69.7 μmol/L. Mean difference between baseline and lowest 7 day follow-up creatinine was significantly lower in the PR AKI group than in I AKI group (*p* < 0.001) (Fig. 2c).

**Fig. 3:**
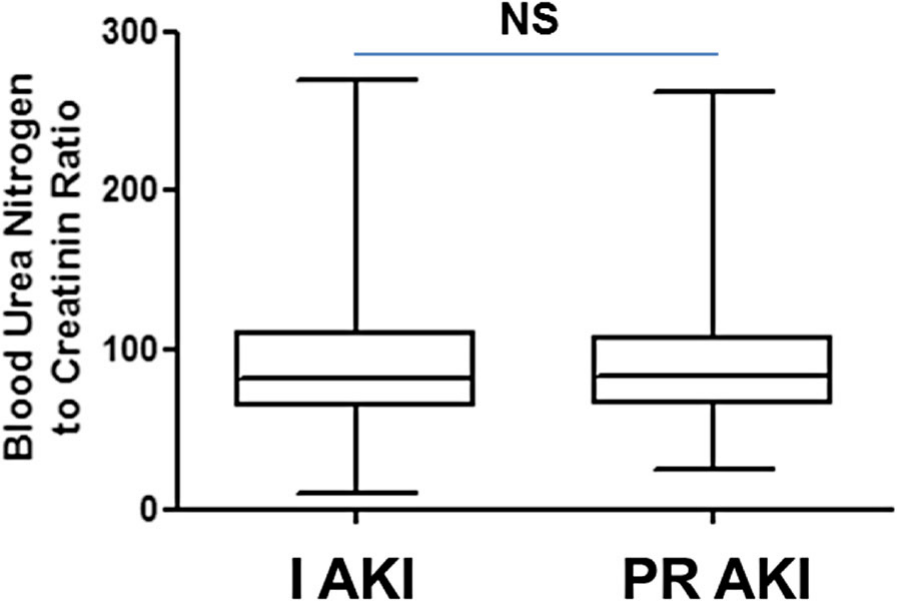
Distribution of blood urea nitrogen to creatinine ratio (BCR) according to AKI groups. The line in the middle of the box is plotted at the median and whiskers delimit min to max values

The distribution of the blood urea nitrogen to creatinine ratio (BCR) in the 2 groups is shown in Fig. 3. At admission, BCR was 90.6 ± 39.3 (range [24.4–262.7]; median 83.0) and 91.3 ± 39.8 (range [9.8–269.1]; median 81.8) in PR AKI and I AKI groups respectively. There was no statistical difference between mean BCR of the prerenal AKI group and the intrinsic AKI group, *p* = 0.758 (Fig. 3 and Table 2).

**Fig. 4:**
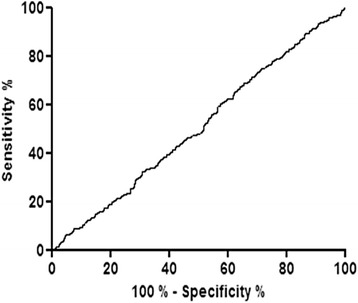
Receiver operating curve analysis of predictive performance of blood urea nitrogen to creatinine ratio (BCR)

The prediction of the capacity of BCR to correctly classify AKI as intrinsic or prerenal was further tested by a Receiver Operating Characteristic (ROC) curve. The area under the curve was 0.5 indicating that the BCR had no capacity to discriminate between prerenal and intrinsic AKI (Fig. 4). At the threshold of 100 commonly used to distinguish PR AKI (BCR > 100) from I AKI (BCR < 100), the sensitivity was 70.1% and the specificity 32.6%.

The distribution of patients with a BCR < 100 or >100 was further analysed and compared to renal recovery (Table 4).

**Table 4:** Distribution of prerenal and intrinsic AKI patients according to whether BCR < or > 100

	BCR < 100	BCR > 100
Number of patients (%)	754 (68.4)	349 (31.6)
PR AKI (%)	45.9	43.6
I AKI (%)	54.1	56.4

If prerenal AKI was redefined as a return of serum creatinine to 110% of baseline in 72 h (rather than 7 days), 418 (38%) patients could be analysed. These are the patients that had BUN and creatinine measured precisely at 72 h. Then 166 (39.7%) patients had PR AKI and 252 (60.3%) I AKI. At admission, mean BCR was 88.4 ± 39.6 (range [24.4–229.8]; median 82.8) and 95.3 ± 42.7 (range [9.8–269.1]; median 86.2) in PR AKI and I AKI groups respectively. There was no statistical difference between mean BCR of the prerenal AKI group and the intrinsic AKI group, *p* = 0.094. The area under the ROC curve was 0.55.

Analysis of patients presenting with acute heart failure (*n* = 191), typically associated with increased BCR, showed that 33% had PR AKI and 67% had I AKI, mean BCR was 98.3 ± 38 (range [37.3–250.6]; median 90.2).

## Discussion

Our study is the largest concerning the diagnostic performance of BCR for differentiating prerenal from intrinsic AKI. It is the first to specifically investigate BCR in an unselected population of patients admitted to the Emergency Department. We have found that BCR had no overall discriminative capacity in this setting, no matter what threshold of BCR is chosen.

Even though the rationale underlying the use of BCR is seducing, our results show that it simply doesn’t work in the real world. Indeed, many factors are known to modify BCR independently of effective circulating volume. Gastro intestinal bleeding, a high protein diet, the catabolic effects of fever, trauma, infection, thyrotoxicosis, drugs such as tetracycline or corticosteroids, all increase protein turnover resulting in increased hepatic production of urea and increase BCR [5, 6]. Conversely, in osmotic diuresis and with the use of acetazolamide, proximal tubular reabsorption of salt and water is impaired leading to an increase in excreted urea and a decrease in BCR even in states of hypovolemia. BCR also decreases in patients with liver failure or protein malnutrition due to lower levels of BUN [5, 6]. One can also speculate that AKI is probably due to functional and intrinsic disease coexisting in different proportions, in a given patient at a given time. Indeed, it is assumed that a continuum exists which leads from prerenal to intrinsic AKI, the proportion of each changing over time [14]. It can then easily be assumed that BCR would only be reliable if the underlying disease was 100% prerenal or 100% intrinsic, which is probably rarely the case.

We chose a “cut off creatinine” for inclusion of patients at 133 μmol/L. The reason for this was twofold: 1/ This threshold is recognized as the highest creatinine level one can have with a normal glomerular filtration rate (GFR) ie 75 mL/min per 1.73m^2^ [15]. Indeed, a young (20–29 years old) black male with a creatinine of 133 μmol/L would have a normal GFR [15]. However, all creatinine levels above 133 μmol/L are associated with altered GFRs. By including only patients with creatinine levels greater than 133 μmol/L we were sure to only include patients with renal failure. 2/ If we had included all 26,229 patients with a measured creatinine, we would have selected many healthy individuals admitted to hospital for the first time. Many patients would have met our exclusion criteria because of a short hospital stay (< 48 h) and or no baseline creatinine.

A limitation that affects most studies on AKI, is the absence of a baseline creatinine for all patients. Indeed, the KDIGO classification scheme requires a baseline creatinine level to define AKI. In the absence of such values a method of “imputation” or “back calculation” by reversing the “Modification of Diet in Renal Disease” (MDRD) equation using age, sex and an assumed normal GFR of 75 mL/min per 1.73m^2^ has been recommended by the ADQI workgroup [16, 17]. This commonly accepted method underestimates baseline creatinine and overestimates GFR [17, 18]. It has been shown that the incidence of AKI actually falls when you rely excessively on the estimation of baseline renal function by this method [12, 19]. Challinger et al. found that 54% of the 745 patients included in their study on AKI in the Emergency Department had no baseline creatinine measurement. These patients were assumed to have a normal estimated GFR. They found an overall incidence of AKI of 24.4%, but this rose to 38.1% if only patients with known previous creatinine records were included [12]. Because of this observation we decided to exclude patients with no creatinine baseline (*n* = 255).

Our methodology induces 2 limitations. Firstly our study was retrospective with the well-known limitations of such studies and in particular it was not possible for us to assess the contribution of the “urine output”, component of the KDIGO criteria. Secondly there is no widely accepted definition of prerenal AKI [6]. The common observation is that it is a reversible condition, but diagnostic criteria such as the amount, nature and duration of fluid resuscitation, time frame for reversibility, or target creatinine improvement have not been addressed. We defined prerenal AKI as a return of serum creatinine to 110% (or less) of the baseline value during the 7 days following admission. In their review of AKI, Bagshaw et al. reported that time frames for defining reversibility ie PR AKI, varied considerably from 48 h to 7 days or more and sometimes were not reported at all [8]. The shorter (48-72 h) is the most published time frame but concerns almost exclusively patients from intensive care units, where blood samples are drawn every day which is not necessarily the case for most patients, such as ours, admitted to medical wards [6]. By choosing a 72 h time frame for reversibility we would have had to exclude many patients who didn’t have blood samples precisely on day 3. We did however carry out a subgroup analysis of these patients. We found 418 (38%) patients that had BUN and creatinine measured at 72 h. Again there was no statistical difference between mean BCR of the prerenal and the intrinsic AKI groups, the area under the ROC curve was 0.55.

Separating prerenal from intrinsic AKI remains challenging. No biological marker, whether old school (fractional excretion of sodium or urea, urinary sodium (UNa,) urine-to-plasma creatinine ratio (U:PCr), etc) or new biomarkers such as Cystatin C, urinary neutrophil gelatinase-associated lipocalin (N-GAL), has clearly demonstrated a capacity to correctly classify AKI [6, 14, 20, 21]. On the ED, the importance of taking a complete medical history, obtaining the list of current potentially nephrotoxic medication, assessing hemodynamic status and if required initiating a fluid challenge, still remains “cornerstone” management of AKI.

## Conclusions

Our study is the largest to investigate the diagnostic performance of BCR. We found that BCR was not a reliable parameter for distinguishing prerenal AKI from intrinsic AKI in a population of patients admitted to hospital via the Emergency Department.

## Abbreviations

AKI: Acute kidney injury
BCR: Blood urea nitrogen to creatinine ratio
BUN: Blood urea nitrogen
ED: Emergency department
GFR: Glomerular filtration rate
I AKI: Intrinsic acute kidney injury
KDIGO: Kidney disease improving global outcomes
MDRD: Modification of diet in renal disease
N-GAL: Neutrophil gelatinase associated lipocalin
PR AKI: Prerenal acute kidney injury
ROC: Receiver operating characteristic
